# Group A Streptococcal Disease in Sudden Unexpected Death in Youth in the Pre- and Post-COVID-19 Era

**DOI:** 10.1097/INF.0000000000004775

**Published:** 2025-02-28

**Authors:** Evelien B. van Kempen, Annelotte M. Pries, Emmeline P. Buddingh, Patrycja J. Puiman, Mirjam van Veen

**Affiliations:** From the * Department of Paediatrics, ErasmusMC Sophia Children’s Hospital, Rotterdam, the Netherlands; † Department of Paediatrics, Juliana Children’s Hospital Haga Hospital, the Hague, the Netherlands; ‡ Department of Paediatrics, Willem-Alexander Children’s Hospital Leiden University Medical Center, Leiden, the Netherlands; § The PESUDY and COPP-iGAS collaborative members are listed in the Appendix.; Department of Paediatrics Maastricht University Medical Center, Maastricht, the Netherlands; Department of Paediatrics, Amsterdam University Medical Center Emma Children’s Hospital, Amsterdam, the Netherlands; Department of Paediatrics, Radboud University Medical Center Amalia Children’s Hospital, Nijmegen, the Netherlands; Department of Paediatrics, Maastricht University Medical Center, Maastricht, the Netherlands; Department of General Paediatrics, Erasmus University Medical Center Sophia Children’s Hospital, Rotterdam, the Netherlands; Department of Paediatrics, University Medical Center Utrecht Wilhelmina Children’s Hospital, Utrecht, the Netherlands; Department of Paediatrics, Canisius Wilhelmina Ziekenhuis, Nijmegen, the Netherlands; Department of Paediatrics, University Medical Center Groningen Beatrix Children’s Hospital, Groningen, the Netherlands; Department of Paediatrics, Leiden University Medical Center Willem-Alexander Children’s Hospital, Leiden, the Netherlands; Department of Paediatrics, Juliana Children’s Hospital HagaHospital, the Hague, the Netherlands; Department of Paediatrics, Willem-Alexander Children’s Hospital Leiden University Medical Center, Leiden, the Netherlands; Department of Paediatrics, Juliana Children’s Hospital HagaHospital, the Hague, the Netherlands; Department of Paediatrics, Willem-Alexander Children’s Hospital Leiden University Medical Center, Leiden, the Netherlands; Department of Paediatrics, Willem-Alexander Children’s Hospital Leiden University Medical Center, Leiden, the Netherlands; Department of Paediatrics, Albert-Schweitzer Hospital, Dordrecht, the Netherlands; Department of Paediatrics, Amsterdam University Medical Center Emma Children’s Hospital, Amsterdam, the Netherlands; Department of Medical Microbiology and Infection Prevention, Amsterdam University Medical Center, Amsterdam, the Netherlands; Department of Paediatrics, Bernhoven Hospital, Uden, the Netherlands; Department of Paediatrics, Haaglanden Medical Center, the Hague, the Netherlands; Bokken (Department of Paediatrics, Maxima Medical Center, Veldhoven, the Netherlands; Department of Paediatrics, Radboud University Medical Center Amalia Children’s Hospital, Nijmegen, the Netherlands; Department of Paediatrics, Northwest Hospital Group, Alkmaar and Den Helder, the Netherlands; Department of Paediatrics, Radboud University Medical Center Amalia Children’s Hospital, Nijmegen, the Netherlands; National Institute for Publich Health and the Environment; Department of Pediatrics, Maasstad Hospital, Rotterdam, the Netherlands; Department of Pediatrics, Division of Pediatric Infectious Diseases and Immunology, Erasmus MC-Sophia Children’s Hospital, University Medical Center Rotterdam, Rotterdam, the Netherlands; Department of General Paediatrics, Erasmus University Medical Center Sophia Children’s Hospital, Rotterdam, the Netherlands; Department of Paediatrics, Spaarne Hospital, Haarlem, the Netherlands; Department of Paediatrics, Sint-Franscisus Gasthuis Hospital, Rotterdam, the Netherlands; Department of Paediatrics, Slingeland Hospital, Doetinchem, the Netherlands; Department of Paediatrics, TerGooi Hospital, Hilversum, the Netherlands; Department of Paediatric Infectious Diseases and Immunology, University Medical Center Utrecht Wilhelmina Children’s Hospital, Utrecht, the Netherlands; Department of Paediatrics/Infectious Diseases, University Medical Center Groningen Beatrix Children’s Hospital, Groningen, the Netherlands; Department of Paediatrics, Zaans Medical Center, Zaandam, the Netherlands

**Keywords:** group A streptococcus, sudden death, epidemiology

## Abstract

**Background:**

An upsurge in pediatric invasive group A streptococcal infection (iGAS) has been observed in the Netherlands along with a suspected increase in iGAS-related sudden death. Sudden unexplained deaths in youth (SUDY) are investigated nationally through a standardized procedure [(Postmortem Evaluation of Sudden Unexplained Death in Youth (PESUDY)]. We investigate epidemiological differences between pediatric iGAS-related sudden deaths (iGAS-PESUDY) and surviving iGAS cases.

**Methods:**

This observational study used data from the COPP-iGAS study on pediatric iGAS infections in Dutch hospitals and the PESUDY database. Children 0–18 years of age were included between August 2016 and December 2022.

**Results:**

Twenty-one iGAS-PESUDY cases and 156 iGAS survivors were included. iGAS-PESUDY cases tended to be older compared to survivors. iGAS-PESUDY cases significantly increased in 2022 compared to the pre-COVID period. Pre- and/or coinciding infections were present in 66% of iGAS-PESUDY cases, predominantly varicella zoster (19%) and influenza (24%). In survivors, 13% had varicella zoster virus and 3% had influenza virus (*P* ≤ 0.001). C-reactive protein levels tended to be lower in iGAS-PESUDY cases (81 mg/L; interquartile range, 26.8–307.5) compared to survivors (266 mg/L; interquartile range, 218.0–302.0).

**Conclusion:**

iGAS is currently a prevalent cause of SUDY. The finding of moderately elevated C-reactive protein levels compared to high levels in survivors might suggest children dying suddenly of iGAS have a rapid and fulminant disease course. Children with a pre- and/or coinciding infection of varicella zoster or influenza virus may be at greater risk of succumbing to iGAS infections.

In the post-COVID-19 pandemic era, there has been a worldwide surge of invasive group A streptococcal infections [invasive group A streptococcal infection (iGAS)] in children, including in the Netherlands.^1,2^ This surge has surpassed the previously observed increasing incidence of iGAS in several countries during the decade prior to COVID-19.^3–5^ Although observations vary per country, a shift in the severity of clinical presentation and possible worsening outcomes have been noted when comparing the pre- and post-COVID-19 pandemic periods.^1,6,7^ *Streptococcus pyogenes*, often synonymously referred to as group A streptococcus (GAS), can cause infections ranging from asymptomatic carriage to noninvasive and invasive infections. Examples of noninvasive infections are tonsillitis, scarlet fever and impetigo, while iGAS infections include pneumonia, sepsis, streptococcal toxic shock syndrome, necrotizing fasciitis, osteomyelitis and meningitis.

The cause of this increase and clinical shift is probably multifactorial, influenced by the implementation of nonpharmaceutical interventions during the COVID-19 pandemic. Decreased exposure to infectious diseases due to COVID-19-related measures to reduce the spread of SARS-CoV-2 may have resulted in an “immunity gap” in children.^8^ Moreover, a post-pandemic increase in cocirculating viruses has been observed, predisposing children to secondary bacterial infections, including GAS.^6,7^ The emergence of more virulent GAS strains may also have contributed.^9,10^

Pediatric iGAS infections pose a significant public health concern as they can cause serious morbidity and rapidly turn fatal. Investigations into sudden death in youth in the Netherlands alerted clinicians to a possible increase in GAS-related deaths post-COVID-19.^11^ These deaths were examined through the Dutch Postmortem Evaluation of Sudden Unexplained Death in Youth (PESUDY) procedure, which aims to determine the cause of death in suddenly deceased children using extensive diagnostic investigations, including microbiological testing of blood, liquor, nasopharyngeal swabs, urine and feces. In 71% of cases, the PESUDY procedure identifies a probable cause of death.^12^

There is a pressing need to understand iGAS-related sudden childhood death to enhance early recognition of fatal iGAS infection, management and development of preventive strategies. The confluence of the heightened incidence of iGAS in children in the post-COVID-19 era and the increasing number of iGAS-PESUDY cases has spurred our research into iGAS-related sudden unexpected childhood fatalities. Our aim was to quantify the proportion of iGAS-related sudden deaths among PESUDY cases and compare these cases to children who survived iGAS infection. We aimed to identify epidemiological factors in iGAS-PESUDY cases that differentiated them from iGAS survivors. These data can provide valuable information to increase earlier identification of fatal iGAS infections and potential interventions.

## METHODS

This is an observational study, comparing iGAS-PESUDY cases and iGAS survivors, with data collected from the national PESUDY database and the COPP-iGAS study database, respectively. Ethical approval was obtained from the appropriate institutional review board for both the collection of data on the PESUDY procedure (MEC-2021-0421/A-0001) and the COPP-iGAS study (METC N20.043).

### iGAS-PESUDY Cases

The Dutch PESUDY database collects data from children investigated through the ongoing PESUDY procedure.^12^ Children 0–18 years of age are eligible for the procedure when they die suddenly, unexpectedly and without a clear cause of death or signs of an unnatural cause. Cases of stillbirth or neonatal death in children never discharged home are excluded. No strict time definition for “suddenly” is used. The complete PESUDY procedure consists of a standardized extensive medical history, postmortem physical examination, biochemical analysis, microbiological analysis, radiologic examinations and an autopsy. The cause of death in PESUDY cases is determined in a multidisciplinary audit with representatives from various fields involving, but not limited to, general pediatrics, pediatric cardiology, pediatric infectious diseases, pediatric metabolic disorders, forensic medicine, radiology, pathology, genetics and sudden infant death syndrome. The database documents detailed patient characteristics, circumstances of the death, diagnostics conducted and outcomes of the PESUDY procedure using standardized data collection forms. iGAS infections were identified through cultures of normally sterile body sites. The primary infectious agent and potential pre- and/or coinciding infection are both determined by the conclusions drawn during the multidisciplinary audit regarding the specific cause of death. Cases from the PESUDY database were included in our study if their parents or legal caregivers consented to at least one part of the PESUDY procedure and use of data for research purposes.

### Surviving iGAS Cases

Surviving cases of iGAS infections were identified through the COPP-iGAS study database.^13^ This is a national, multicenter, observational, retrospective and prospective cohort study conducted in the Netherlands. Twenty hospitals throughout the Netherlands participated, including all 7 Dutch academic hospitals with pediatric intensive care units. Children 0–18 years old were included between January 2015 and June 2024 after in-hospital diagnosis with iGAS (emergency department presentation or hospitalization). An iGAS diagnosis was established on one of the following criteria: (1) positive culture or molecular detection [polymerase chain reaction (PCR)-based methods, eg, 16S ribosomal RNA] for GAS in a normally sterile body site, such as blood, cerebrospinal fluid, pleural fluid or synovial fluid; (2) clinical diagnosis of streptococcal toxic shock syndrome or necrotizing fasciitis; OR (3) positive molecular detection or culture for GAS from a nonsterile body site without another pathogen detected, alongside a clinical picture compatible with iGAS. Patients were retrospectively identified through the hospital’s local microbiological database systems and/or prospectively by the local investigator.

Data on case characteristics, outcomes and diagnoses are routinely collected and documented using standardized forms. The data were extracted from the COPP-iGAS database on January 2, 2024. At the time of this data retrieval, 11 hospitals had contributed data. Varicella zoster virus infection was diagnosed clinically, while other confirmed pre- and/or coinciding infections were identified through PCR; however, such testing was not routinely performed.

We included all PESUDY cases and iGAS survivors from the respective databases between October 2016 and December 2022. The study periods were categorized as follows: the pre-COVID-19 pandemic period (2016–2019), the COVID-19 pandemic period (2020 and 2021) and the post-COVID-19 pandemic period (2022).

Abnormal temperature was defined as hypothermia or fever measured at presentation (<36°C or >38°C) or reported by parents in the day and/or week before.

Descriptive and statistical analysis of the quantitative data using ^−2^-square, Fisher exact and Mann-Whitney *U* tests for nonparametric data were performed using SPSS (IBM Corp. Released 2021. IBM SPSS Statistics for Windows, Version 28.0. Armonk, NY: IBM Corp).

## RESULTS

**FIGURE 1.**
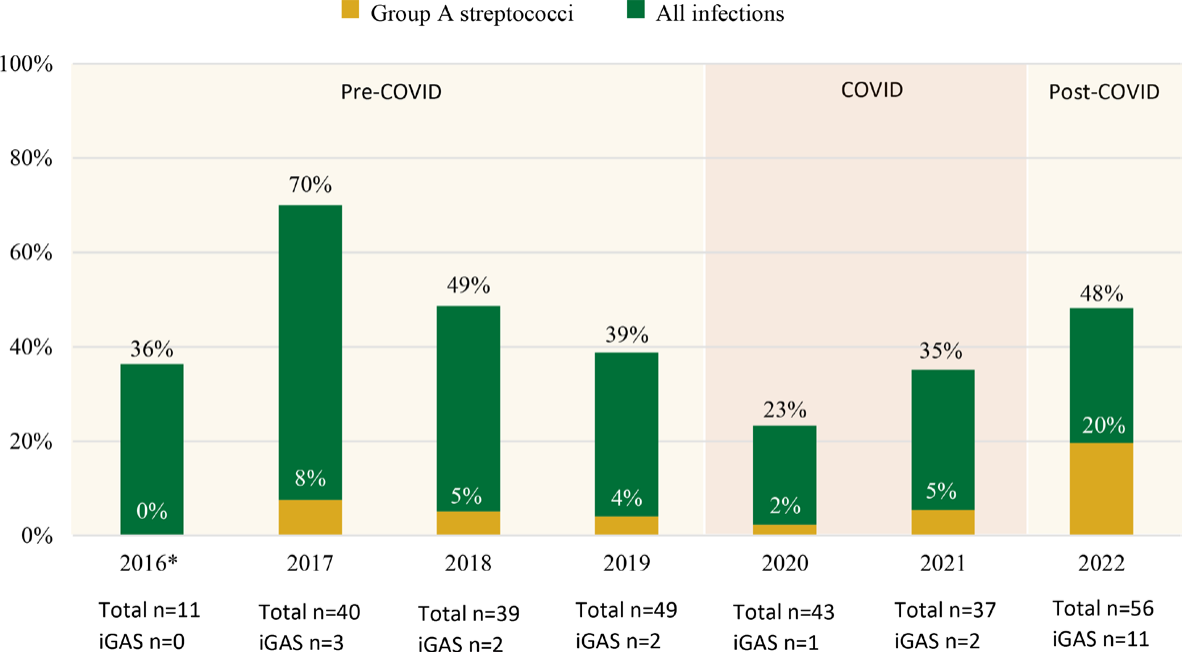
Percentage of total infectious and iGAS-PESUDY deaths as a proportion of the total number of PESUDY cases pre-COVID, during COVID and post-COVID. *Data available from October 2016 until December 2016.

Between 2016 and 2022, a total of 275 deceased children were investigated through the PESUDY procedure. Among these, 21 cases (8%) were determined to be caused by iGAS. Other microbiological agents were identified less frequently. All iGAS-related deaths had at least 1 positive culture or PCR for GAS from a normally sterile body compartment. The incidence of iGAS-related PESUDY deaths increased significantly in the post-COVID period compared to the pre-COVID period (odds ratio: 6.2; CI: 2.1–18.5; *P* = 0.001) (Fig. 1). In 2022, iGAS-related deaths accounted for 20% of all investigated PESUDY deaths compared to 0%–8% pre-COVID and COVID years.

For the same study period, we included a total of 156 iGAS survivors from the COPP-iGAS study, all of whom were admitted to the hospital. Data from 47 cases were collected prospectively from 7 regional hospitals and 4 university hospitals with pediatric intensive care unit facilities. Data from 109 cases were collected retrospectively from 3 regional hospitals and 2 university hospitals with pediatric intensive care unit facilities.

Table 1 presents the characteristics of the iGAS-PESUDY cases and the iGAS survivors.

**TABLE 1.** Basic Characteristics of Cases

Characteristic	iGAS-PESUDY, N = 21 (%)	Surviving iGAS cases, N = 156 (%)
Age, median (yr)	2.6 (IQR: 1.5–6.6)	-
Age category (yr)*		
<1	2 (10)	6 (4)
1–6	12 (57)	132 (85)
6–12	4 (19)	6 (4)
≥12	3 (14)	3 (2)
Unknown	0 (0)	9 (6)
Medical help-seeking for symptoms*	17 (81)	156 (100)
Pre- or coinciding infection	14 (66)	-
Varicella Zoster virus	4 (19)	20 (13)
Influenza virus*	5 (24)	5 (3)
Diagnosis/presentation†		
Sepsis*	20 (95)	25 (16)
Pneumonia	6 (29)	24 (15)
Cardiac infection*	1 (5)	0 (0)
Enteritis*	1 (5)	0 (0)
Meningitis*	2 (10)	1 (1)
Urine tract infection	0 (0)	0 (0)
Osteo/articular infection	0 (0)	10 (6)
Abscess	1 (5)	30 (19)
Lymphadenitis	0 (0)	14 (9)
Pyomyositis	0 (0)	1 (1)
Skin infection	0 (0)	23 (15)
Other	0 (0)	24 (15)

Some characteristics were not available for all iGAS survivor cases.

* Significant difference between iGAS-PESUDY and surviving iGAS cases.

† Multiple diagnoses/presentations per case are possible.

The distribution of ages between the groups differs significantly, as 33% of iGAS-PESUDY cases are >6 years old compared with 6% of iGAS survivors. The majority of iGAS-PESUDY cases were girls (67%), whereas 55% of the iGAS survivors were boys. Comorbidities were present in 29% of iGAS-PESUDY cases compared to 36% of surviving iGAS cases. Both groups reported abnormal temperatures before death or presentation.

A pre- and/or coinciding infection was present in 66% of iGAS-PESUDY cases, with 4 cases diagnosed with varicella zoster virus and five with influenza virus, both confirmed by PCR. Among the iGAS survivors, 13% had a pre- and/or coinciding infection with varicella zoster virus, as reported in their history. Influenza virus was identified significantly more in iGAS-PESUDY cases compared to iGAS survivors (24% vs. 3%, *P* ≤ 0.001).

The median C-reactive protein (CRP) level was 81 mg/L (interquartile range: 26.8–307.5) in iGAS-PESUDY cases (n = 10) and 266 mg/L (interquartile range: 218.0–302.0) in iGAS survivors (n = 31), although this difference was not statistically significant (*P* = 0.06). Most children with iGAS-related sudden deaths were diagnosed with a systemic infection (95%, n = 20/21). In contrast, the presentations of iGAS survivors were more diverse, with significantly fewer children diagnosed with sepsis (16%, n = 25/256) (*P* ≤ 0.001).

## DISCUSSION

Our findings indicate that iGAS is the most identified microbiological culprit in cases of Sudden Unexpected Death in Youth (SUDY) in the Netherlands, accounting for 20% of all investigated SUDY cases in 2022. Children aged 6 years or older were overrepresented in the iGAS-PESUDY cases. Moreover, our results show that iGAS-PESUDY cases tended to exhibit lower CRP levels in comparison to iGAS survivors. Additionally, we found a high proportion of pre- and/or coinciding infections in iGAS-PESUDY cases and a significantly higher number of influenza virus infections in contrast to iGAS survivors.

Our study with cases from 2016 to 2022 showed that GAS was responsible for 18% of infection-related sudden deaths and 8% of total sudden deaths. The large increase in iGAS-related sudden deaths seen in our study aligns with global reports of increased iGAS cases and severity in children post-pandemic.^1,13^ Within the PESUDY cohort, no other pathogen achieved such dominance as GAS (unpublished data). Between 2012 and 2016, pediatric iGAS-related mortality was estimated at 2% throughout Europe. The mortality rate for the recent surge in pediatric iGAS seems to vary across European countries.^1,5,14^ However, studies reporting on SUDY pre-COVID reported low numbers of iGAS involvement, with the highest prevalence reported by Kruger of 0.8% in their 2018 systematic review.^15–17^ There are no recent reports available on microbiological causes of sudden death in youth from other countries.

The reasons why some children succumb to iGAS while others survive or remain asymptomatic carriers are complex and not fully understood. Our study observed that sepsis was more prevalent in iGAS-PESUDY cases compared to the surviving iGAS cases (Table 1). This observation might suggest that the rapid disease course in this group of children with sudden and unexpected death might be the consequence of more aggressive disease. Several hypotheses could potentially explain these differences in clinical outcomes of iGAS infections. First, predisposing viral infections may play an auxiliary role in fatal iGAS cases. Second, genetic factors may predispose certain children to a heightened susceptibility to iGAS infections or lead to an exaggerated inflammatory response. Third, the virulence of the GAS strain itself may vary, contributing to differences in disease severity and outcomes among children.^6,9,14^

Our study demonstrated a large proportion of pre- and/or coinciding infections by varicella zoster virus and influenza virus in iGAS-PESUDY cases. Both varicella zoster virus and influenza virus infections are known risk factors that may compromise the immune response or create conditions favorable for GAS to cause severe disease.^18–20^ Epidemiological studies have consistently shown that pre- and/or coinciding (viral) infections of either the upper respiratory tract or skin render the host more vulnerable to bacterial superinfection by disrupting mucosal or skin barriers.^1,7,21^ Alternatively, the presence of a coinciding infection might result in false reassurance and delay in appropriate management.

Both varicella zoster virus and GAS have been implicated in impairing immune function, potentially exacerbating the severity of infections.^18,19,22^ Similarly, it has been suggested that influenza virus infection weakens the efficacy of the immune system to combat bacterial pathogens and impede bacterial clearance.^19,22^ Previous research has shown the benefits of varicella zoster virus or influenza virus vaccination in reducing iGAS incidence but did not investigate prehospital iGAS-related mortality.^23,24^ Considering our findings, investigating the influence of vaccination against varicella zoster and/or influenza virus on iGAS-related sudden death in children might lead to new preventative strategies. Currently, the Dutch immunization program does not include immunization against varicella zoster and influenza vaccination is only recommended for certain risk groups.

It is reasonable to assume that the CRP levels measured in our study reflect antemortem levels. Previous studies have shown that CRP levels in adults tend to remain stable or decrease up to 35% postmortem compared to antemortem analyses.^25,26^ The lower CRP levels observed in iGAS-PESUDY cases could suggest that these children may have succumbed to their infection before experiencing a significant increase in CRP levels. Such a rapid and fatal disease progression could be caused by underlying host immunogenic predisposition leading to increased susceptibility to severe pyogenic infections.^27^ Although overt primary immunodeficiencies seem unlikely in our study’s iGAS-related deaths as these children had low comorbidity and no history of recurrent bacterial infections or poor growth, deficiencies in Toll-like receptor signaling pathways are known to put otherwise healthy individuals at risk for severe pyogenic infections. Examples include IRAK-4 deficiency and MyD88 deficiency. Patients with IRAK-4 or MyD88 deficiency remarkably often do not develop fever and exhibit low levels of inflammation markers, such as CRP.^28^ It is noteworthy that 10%–20% of children with invasive pneumococcal disease have an underlying primary immunodeficiency.^29,30^ The prevalence of immunogenic predisposition has not been studied in iGAS, providing a basis for investigating this hypothesis to better understand our findings.

The final hypothesis is that children who succumbed to iGAS infections may have been infected by a particularly virulent strain of GAS characterized by a specific *emm*-type or genetic clone. *Emm*-typing, a method used to identify specific *emm* types of invasive *GAS* isolates, is performed by the Netherlands Reference Laboratory for Bacterial Meningitis since 2019. As of May 2022, all microbiological laboratories have been requested to submit invasive GAS isolates for further analysis. However, during the timeframe of our study cases, *emm*-typing, or the identification of virulent mutations, was not routinely performed.

Our observations and proposed hypotheses, including viral coinfections, genetic predispositions and bacterial virulence, could provide valuable insights into the mechanisms underlying severe iGAS infections. This understanding may help in designing strategies for early identification, treatment and prevention. Further research is needed to elucidate the specific interactions between these factors and their impact on disease progression in pediatric iGAS cases.

Our study provides a unique comparison between pediatric iGAS-related deaths and surviving iGAS cases, drawing from detailed data sources, including the national PESUDY database for SUDY and the COPP-iGAS study database for pediatric survivors, in a representative region of the Netherlands for the same period. The COPP-iGAS study design limited systemic bias by collecting data from different types of hospitals across the Netherlands, including academic, teaching and smaller regional hospitals, ensuring a broad coverage of clinical presentations, reduction of regional differences and therefore providing a representative sample of Dutch pediatric iGAS patients. Selection bias was also reduced by having microbiological samples as the main identifier for eligible cases, with positive isolates from both sterile and non-sterile compartments. Several limitations should be considered.

One limitation is the retrospective nature of part of our data collection, which introduces the possibility of information bias. Retrospective data collection relies on recorded information, which may be incomplete or inconsistently documented, potentially affecting the accuracy of our findings. Additionally, in the cohort of iGAS survivors, the reporting of pre- and/or coinciding infections relied on clinical diagnosis by attending physicians, rather than routine testing in all cases. This could have led to underreporting of influenza and varicella virus infections in the surviving iGAS cases, thereby potentially skewing our understanding of the prevalence and impact of these infections. There were also no data available on possible differences in caregiver awareness of illness or socioeconomic level between the groups. Another limitation is the exclusion of SUDY cases that were not investigated through the PESUDY procedure. While we estimate this exclusion does not significantly impact our results—given Dutch national death statistics indicating approximately 50 sudden deaths per year and an average of 44 cases included annually in PESUDY—there remains a possibility of selection bias in the cases included in our study. For the surviving iGAS cases, uncomplicated cases of pneumonia might have gone undetected, since only cases with microbiological confirmation of GAS, besides STSS and NF, were included. iGAS in general could have been underdetected in both groups, since no clinically validated blood PCR is available.

Our findings highlight iGAS as the predominant microbiologic cause of SUDY in the Netherlands in the post-COVID era, particularly associated with pre- and/or coinciding infections and older children. Together with the observed discrepancies in CRP levels and prevalence of systemic infections, suggesting a rapid and severe disease progression, these results provide clues for further research into viral coinfections, genetic predispositions and bacterial virulence. Unraveling the complexities of iGAS pathogenesis and clinical variability will aid in developing targeted interventions, improving clinical outcomes and reducing mortality rates in children.

## ACKNOWLEDGMENTS

*The authors thank the Dutch National Health Council (ZonMW) for their support of the COPP-iGAS study.*

## APPENDIX

The members of the PESUDY collaborative include: A. Custers (Department of Paediatrics, Maastricht University Medical Center, Maastricht, the Netherlands), E. Edelenbos (Department of Paediatrics, Amsterdam University Medical Center Emma Children’s Hospital, Amsterdam, the Netherlands), J. Fuijkschot (Department of Paediatrics, Radboud University Medical Center Amalia Children’s Hospital, Nijmegen, the Netherlands), B. Levelink (Department of Paediatrics, Maastricht University Medical Center, Maastricht, the Netherlands), P.J. Puiman (Department of General Paediatrics, Erasmus University Medical Center Sophia Children’s Hospital, Rotterdam, the Netherlands), J.M. Ruskamp (Department of Paediatrics, University Medical Center Utrecht Wilhelmina Children’s Hospital, Utrecht, the Netherlands), B. Semmekrot (Department of Paediatrics, Canisius Wilhelmina Ziekenhuis, Nijmegen, the Netherlands), K.T. Verbruggen (Department of Paediatrics, University Medical Center Groningen Beatrix Children’s Hospital, Groningen, the Netherlands) and H. Vlaardingerbroek (Department of Paediatrics, Leiden University Medical Center Willem-Alexander Children’s Hospital, Leiden, the Netherlands).

The members of the COPP-iGAS collaborative include: Mirjam van Veen (Department of Paediatrics, Juliana Children’s Hospital HagaHospital, the Hague, the Netherlands), Emmeline Buddingh (Department of Paediatrics, Willem-Alexander Children’s Hospital Leiden University Medical Center, Leiden, the Netherlands), Evelien van Kempen (Department of Paediatrics, Juliana Children’s Hospital HagaHospital, the Hague, the Netherlands), Adam Tulling (Department of Paediatrics, Willem-Alexander Children’s Hospital Leiden University Medical Center, Leiden, the Netherlands), Erik von Asmuth (Department of Paediatrics, Willem-Alexander Children’s Hospital Leiden University Medical Center, Leiden, the Netherlands), Ankie Lebon (Department of Paediatrics, Albert-Schweitzer Hospital, Dordrecht, the Netherlands), Merijn Bijlsma (Department of Paediatrics, Amsterdam University Medical Center Emma Children’s Hospital, Amsterdam, the Netherlands), Nina van Sorge (Department of Medical Microbiology and Infection Prevention, Amsterdam University Medical Center, Amsterdam, the Netherlands), Jan van der Linden (Department of Paediatrics, Bernhoven Hospital, Uden, the Netherlands), Jantien Bolt (Department of Paediatrics, Haaglanden Medical Center, the Hague, the Netherlands), Lonneke van Onzenoort-Bokken (Department of Paediatrics, Maxima Medical Center, Veldhoven, the Netherlands), Else Bijker (Department of Paediatrics, Radboud University Medical Center Amalia Children’s Hospital, Nijmegen, the Netherlands), Dorine Borensztajn (Department of Paediatrics, Northwest Hospital Group, Alkmaar and Den Helder, the Netherlands), Kim Stol (Department of Paediatrics, Radboud University Medical Center Amalia Children’s Hospital, Nijmegen, the Netherlands), Brechje de Gier (National Institute for Publich Health and the Environment), Navin Boeddha (Department of Pediatrics, Maasstad Hospital, Rotterdam, the Netherlands; Department of Pediatrics, Division of Pediatric Infectious Diseases and Immunology, Erasmus MC-Sophia Children’s Hospital, University Medical Center Rotterdam, Rotterdam, the Netherlands), Rianne Oostenbrink (Department of General Paediatrics, Erasmus University Medical Center Sophia Children’s Hospital, Rotterdam, the Netherlands), Marlies van Houten (Department of Paediatrics, Spaarne Hospital, Haarlem, the Netherlands), Gerdien Tramper (Department of Paediatrics, Sint-Franscisus Gasthuis Hospital, Rotterdam, the Netherlands), Monique Jacobs (Department of Paediatrics, Slingeland Hospital, Doetinchem, the Netherlands), Caroline Kosterink-Brackel (Department of Paediatrics, TerGooi Hospital, Hilversum, the Netherlands), Joanne Wildenbeest (Department of Paediatric Infectious Diseases and Immunology, University Medical Center Utrecht Wilhelmina Children’s Hospital, Utrecht, the Netherlands), Aline Verhage (Department of Paediatrics/Infectious Diseases, University Medical Center Groningen Beatrix Children’s Hospital, Groningen, the Netherlands) and Leontien van der Aa (Department of Paediatrics, Zaans Medical Center, Zaandam, the Netherlands).
